# Association of common variants in JAK2 gene with reduced risk of metabolic syndrome and related disorders

**DOI:** 10.1186/1471-2350-12-166

**Published:** 2011-12-20

**Authors:** Alberto Penas-Steinhardt, Mariana L Tellechea¹, Leonardo Gomez-Rosso, Fernando Brites, Gustavo D Frechtel, Edgardo Poskus

**Affiliations:** 1 Humoral Immunity Institute "Prof. Ricardo A. Margni" (IDEHU), National Research Council (CONICET) & Chair of Immunology, School of Pharmacy and Biochemistry, University of Buenos Aires (UBA) - Argentina; 2 Chair of Genetic, Department of Microbiology, Inmunology and Biotecnology, School of Pharmacy and Biochemistry, University of Buenos Aires (UBA) - Argentina; 3 Laboratory of Lipids and lipoproteins, Department of Clinical Biochemistry, School of Pharmacy and Biochemistry. National Research Council (CONICET) & University of Buenos Aires (UBA) - Argentina; 4 Genetics Division, Clinical Hospital "José de San Martín", University of Buenos Aires (UBA) - Argentina

## Abstract

**Background:**

Disturbances in leptin and insulin signaling pathways are related to obesity and metabolic syndrome (MS) with increased risk of diabetes and cardiovascular disease. Janus kinase 2 (JAK2) is a tyrosine kinase involved in the activation of mechanisms that mediate leptin and insulin actions. We conducted a population cross-sectional study to explore the association between two common variants in JAK2 gene and MS related traits in 724 Argentinean healthy male subjects.

**Methods:**

A total of 724 unrelated men aged 37.11 ± 10.91 yr were included in a cross-sectional study. Physical examination, anthropometric measurements and biochemical analysis were determined by a standardized protocol. rs7849191 and rs3780378 were genotyped. Analyses were done separately for each SNP and followed up by haplotype analysis.

**Results:**

rs7849191 and rs3780378 were both associated with reduced risk of MS [p = 0.005; OR (95%CI) = 0.52 (0.33-0.80) and p = 0.006; OR (95% CI) = 0.59 (0.40-0.86) respectively, assuming a dominant model]. rs3780378 T allele was associated with triglyceridemia values under 150 mg/dl [p = 0.007; OR (95%CI) = 0.610 (0.429-0.868)] and TT carriers showed lower triglycerides (p = 0.017), triglycerides/HDL-C ratio (p = 0.022) and lipid accumulation product (p = 0.007) compared to allele C carriers. The two-SNPs-haplotype analysis was consistent with single locus analysis.

**Conclusions:**

It was found for the first time, significant associations of JAK2 common variants and related haplotypes with reduced risk of MS. These findings could be explained by the role of JAK2 in insulin and/or leptin signaling.

## Background

The metabolic syndrome (MS) is a constellation of cardiometabolic risk factors including central obesity, insulin resistance (IR), hypertension, hyperglycemia, and dislipidemia [1,2]. Insulin resistance (IR) is probably the principal cause of the MS [3,4]. Over the past decades, a sustained worldwide increase in the incidence of MS has taken place, associated with the global epidemic of obesity and type 2 Diabetes Mellitus (DM2) [5].

Janus kinase 2 (JAK2) is a nonreceptor tyrosine kinase recruited by receptors that lack intrinsic kinase activity [6]. The JAK/STAT (signal transducer and activator of transcription) pathway transmits a wide range of cytoplasmic signals from cytokines, growth factors and hormones that bind to specific cell surface receptors [7]. Suppressor of cytokine signaling (SOCS) proteins are known to act as negative regulators of cytokine action via inhibition of these pathways. There is a growing body of evidence that supports a role for specific SOCS members in the development of IR [8].

Insulin and leptin are major signals to regulate energy homoeostasis and body adiposity [9,10]. After binding to its receptor, leptin induces activation of JAK2 and subsequent phosphorylation of specific tyrosines on the receptor [11]. The cytokine-like hormone leptin receptor is a member of the class I cytokine receptor family found in many tissues in several alternatively spliced forms [12,13]. The long isoform (LepRb) transduces signal mainly through the JAK2/STAT3 pathway. JAK2 also phosphorylates Insulin Receptor Substrates 1 and 2 (IRS-1 and IRS-2) integral to insulin and leptin action [14]. Insulin carries out its biological effects through the phosphorylation of IRS-1 and IRS-2 and it is also well documented the cross-talk between the insulin and leptin signal transduction pathways. Leptin was reported to induce IRS-1 and IRS-2 phosphorylation [15].

Recently, although not significant at levels which took account of multiple testing, suggestive associations between two single nucleotide polymorphisms (SNPs) of JAK2 gene and central fat, waist circumference and serum lipid variables have been reported [16]. Replication is fundamental for deciding that an observed association is likely not due to chance [17]. Therefore, the aim of this study was to explore associations between these two SNPs of JAK2 and their predicted haplotypes on MS and related phenotypes and quantitative metabolic traits.

## Methods

### Subjects

The total sample includes 724 unrelated men of self-reported European ancestry, especially from Spain and Italy, living in Buenos Aires metropolitan area. Individuals were selected from a cross-sectional study. Participants were recruited, between April 2006 and April 2008, from the Departement of Haemotherapy of the Hospital "José de San Martín" of the University of Buenos Aires. Ages ranged between 18 to 65 years. Clinical characteristics of the sample are shown in Table 1. All subjects were non diabetic with normal findings on medical examination and blood count. This work was carried out in accordance with the Declaration of Helsinki, and approved by the Ethic Committee of our Hospital. All subjects gave their written consent previously to participate in the study (clinical characteristics are summarized in Table 1).

**Table 1:** Clinical characteristics of the sample

	Mean	SD	Phenotype	Prevalence (%)
Age (yr)	37.11	10.91	HTG	31.1
BMI (kg/m2)	28.20	4.40	HW	27.8
WC (cm)	96.15	12.26	Decreased HDL-C	45.6
SBP (mmHg)	126.29	11.02	Abdominal obesity	31.3
DBP (mmHg)	80.11	7.38	Obesity	29.3
TC (mg/dl)	187.45	40.75	IFG	18.0
LDL-C (mg/dl)	118.52	34.89	MS	24.1
HDL-C (mg/dl)	41.25	10.66		
TG (mg/dl)	138.77	95.97		
LAP	52.74	6.72		
TG/HDL-C	3.86	3.73		
FPG (mg/dl)	91.87	12.23		
Fasting insulin	16.18	9.27		
HOMA-IR	2.07	1.16		

n = 724. DBP, diastolic blood pressure; FPG, fasting plasma glucose; HDL-C, high-density lipoprotein cholesterol; HOMA-IR, homeostasis model assessment of insulin resistance; HW, hypertriglyceridemic waist; IR, insulin resistance; LDL-C, low-density lipoprotein cholesterol; SBP, systolic blood pressure; TC, total cholesterol; TG, triglycerides; LAP = lipid accumulation product; WC, waist circumference; HTG = Hypertriglyceridemia; HW = hypertriglyceridemic waist; IFG = impaired fasting glucosa; MS = metabolic syndrome

### Clinical measurements

Anthropometric measurements (height, weight and waist circumference) and Systolic (SBP) and Diastolic (DBP) blood pressure were determined by a standardized protocol in every subject.

After a 12-h overnight fast, fasting blood samples were drawn in every individual from an antecubital vein. Total cholesterol (TC), triglycerides (TG), and high-density lipoprotein (HDL-C) were determined in serum by enzymatic methods using commercial kits (Roche Diagnostics, Mannheim, Germany) in a Hitachi 727 autoanalyzer. All determinations were performed using standardized procedures. Intra assay-CV (coefficient of variation) for TG and total cholesterol were 1.3 and 1.1%, respectively. Inter assay-CVs were 2.4% and 1.5%, respectively. Fasting plasma glucose (FPG) was determined by a glucose-oxidase method (Roche Diagnostics, Mannheim, Germany) in a Hitachi 727 autoanalyzer. Intra- and inter-CVs were 0.9 and 1.8%, respectively. Fasting serum insulin was measured by radioimmunoassay with a commercial kit (Human Insulin Specific RIA kit, Linco Research Inc., St. Louis, MO, USA) by counting on a gamma counter (DPC Gambyt CR, Los Angeles, USA), with a lower detection limit of 2 μU/ml, intra- and inter-CVs being < 1 and < 7.43%, respectively. Cross-reactivity was less than 0.2% to intact human proinsulin.

### Calculations

Insulin sensitivity was assessed with Homeostasis Model Assessment of insulin resistance (HOMA-IR) using the software HOMA Calculator v.2.2.2 for Windows [18] Low-density lipoprotein cholesterol (LDL-C) was calculated using the Friedewald formula [19]. In addition, TG/HDL-C ratio and lipid accumulation product (LAP) were calculated. LAP, an index of lipid overaccumulation associated with cardiovascular risk and identification of diabetes, was defined (for men) as (waist circumference (WC) [cm] - 65) × (TG concentration [mmol/L]) [20].

Each subject was assessed for the presence of 1) Obesity according to body mass index (BMI) ≥ 30.0 kg/m^2 ^[21]; 2) abdominal obesity according to WC > 102 cm [22]; 3) hypertriglyceridemia (HTG) according to fasting TG ≥150 mg/dl or drug treatment; 4) hypertriglyceridemic waist (HW) according to TG ≥ 150 mg/dl + WC ≥ 90 cm; 5) decreased HDL-C according to fasting HDL-C < 40 mg/dl or drug treatment; 6) Impaired Fasting Glucose (IFG) according to Fasting Plasma Glucose (FPG) ≥ 100 mg/dl; and 7) MS using the American Heart Association/National Heart, Lung, and Blood Institute (AHA/NHLBI) 2005 criteria [23]. Participants who had FPG ≥ 126 mg/dl were excluded from the study [24].

### SNP selection and genotyping

Since replication of findings is an important requirement for genetic association studies, we studied two SNPs (rs7849191 and rs3780378) based on a previous report of Ge et al [16]. The linkage disequilibrium (LD) analysis (Haploview 4.1 [25]) of the 142.8 kb region under study showed that both SNPs capture 20% of alleles with minor allele frequency (MAF) ≥ 10% at r^2 ^≥ 0.8 [we downloaded information from The International HapMap Project web site (http://www.hapmap.org) (HapMap Data Rel 28 PhaseII+III, August10) from the Caucasian European Utah dataset (CEPH)]. Of note, rs7849191 is not in LD with other SNPs and rs3780378 is in LD with other 17 SNPs (rs2230724, rs4372063, rs10815144, rs7037207, rs2149556, rs1328918, rs7034753, rs7875908, rs10115312, rs7023146, rs7857730, rs7847294, rs6476939, rs7032785, rs7043371, rs1328917, rs3780378).

Genomic DNA was isolated from peripheral blood according to standard procedures. The two SNPs were genotyped by KBiosciences, Hoddesdon, Herts., UK, using the KASPar system. This is a fluorescence based allele-specific PCR procedure with improved robustness and discriminating power over conventional ARMS, (http://www.kbioscience.co.uk/chemistry/chemistry-intro.htm). Genotyping accuracy was assessed by inclusion of duplicates and negative controls. Genotyping success rate was 100% for rs7849191 and 98.7% rs3780378. Our lab randomly sampled 10% of the subjects for sequencing to corroborate the initial findings. Using these criteria consistency was 100%.

For each individual case-control study power estimations were performed for single-point allelic effects, with an odds ratio of 1.5 at a nominal significance level of 0.05 [26]. Power estimation was found to be less than 80% to detect effects on MS. However, to detect effects on HTG, HW, obesity or abdominal obesity, power estimation was found to be between 81 and 99% assuming a dominant model and less than 80% assuming a recessive model for rs7849191; and between 80 and 98% assuming a recessive model and less than 80% assuming a dominant model for rs3780378. The power estimation was found to be between 85 and 99% to detect effects of rs7849191 and rs3780378 on decreased HDL-C.

### Statistical analysis

Deviation of the genotype distribution from the Hardy-Weinberg equilibrium (HWE) was tested using Chi square (χ^2^) test. We calculated odds ratio (OR) and 95% confidence intervals (95%CI). Analyses were done separately for each SNP and followed up by haplotype analysis. For individual SNP the statistical difference in genotype distribution and allele frequencies among the groups for qualitative variables was assessed by the Fisher exact test. Binary logistic regression was used to adjust for possible confounding variables. Quantitative data were expressed as means ± SD. For comparison of continuous variables we conducted one-way ANOVA with Levine's Test for equality of variances. Otherwise, we used Welch tests. Multiple linear regression analysis was used to adjust for possible confounding variables. Furthermore, genotype status and age subgroup (2 subgroups according to the average age: < 37 and ≥37 years old) were used as factors in a Multivariate linear regression analysis to determine interaction of age and genotype.

We reconstructed the two SNP -haplotypes using PHASE 2.1 [27]. The haplotype frequencies were estimated using the programs PHASE 2.1 and Thesias [28,29] We analyzed haplotypic effects using the Thesias software and conventional statistical methods as well. A Fisher exact test was used to compare haplotype frequencies. The effects of a particular haplotype load (0: no copies of the particular haplotype; 1: 1 copy; and 2: 2 copies) on continuous variables were tested using a linear regression.

A two sided p-value less than 0.05 was considered statistically significant. Since our results represent a basic replication of previously reported findings, p-values were not corrected for the number of tests performed. However, we follow the recommendations of van den Oord and Sullivan [30], which suggest that a level of significance of p = 0.01 on average control the false discovery rate at 0.10. Note that after applying the Bonferroni correction for multiple tests, the significance level was p < 0.025 (0.05/2 for 2 loci). The significance level was p < 0.01 (0.05/4) for correction for multiple tests in haplotype analyses (4 haplotypes for 2 loci). Statistical analyses were conducted using the program for Statistical Package for the Social Sciences, version 12.0 for Windows (SPSS, Inc., Chicago, IL).

## Results

Both rs7849191 (χ^2 ^= 1.52, p = 0.22) and rs3780378 (χ^2 ^= 0.086, p = 0.77) were in HWE. Genotypic frequencies were as follow: rs7849191: CC 0.40 CT 0.45 and TT 0.15; and rs3780378: TT 0.26 TC 0.49 and CC 0.25. The MAFs were 0.037 and 0.49 for rs7849191 and rs3780378 respectively, similar to previously reported in Caucasians (http://www.ncbi.nlm.nih.gov/snp/).

### Single-Locus Analyses

#### Association with MS

SNPs rs7849191 and rs3780378 were both associated with MS. Age adjusted association between rs7849191 and MS was not significant, but association between rs3780378 and MS remained significant taking into account the effects of age (p = 0.002). C allele of rs7849191 and T allele of rs3780378 showed a lower risk for MS assuming both dominant and recessive model. Adjusted analysis confirmed these associations (Table 2). Note that associations with MS with both rs7849191 and rs3780378 under a dominant model, even accounting for the effects of covariates, were significant at levels that take into account multiple testing.

**Table 2:** Association between individual JAK2 SNPs, Metabolic Syndrome and related phenotypes

	MS negative	MS positive	OR (IC 95%)	p	**p**^a^	**p**^b^
**rs7849191**						
TT vs. TC vs. CC	69 vs. 241 vs. 226	38 vs. 76 vs. 57	-	0.009	ns	ns
CC + CT vs. TT	467 vs. 69	133 vs. 38	0.517 (0.333-0.803)	0.005	0.001	0.002
CC vs. CT + TT	226 vs. 310	57 vs. 114	0.686 (0.478-0.984)	0.048	0.011	0.034
**rs3780378**						
TT vs. TC vs. CC	149 vs. 269 vs. 121	33 vs. 83 vs. 57	-	0.008	0.002	ns (0.096)
TT +TC vs. CC	418 vs. 121	116 vs. 57	0.589 (0.404-0.858)	0.006	0.001	0.007
TT vs. TC+ CC	149 vs. 390	33 vs. 140	0.617 (0.404-0.942)	0.027	0.020	0.022
	**HTG negative**	**HTG positive**	**OR (IC 95%)**	**p**	**p**^a^	**p**^b^
**rs3780378**						
TT vs. TC vs. CC	139 vs. 250 vs. 108	48 vs.108 vs. 71	-	0.013	0.006	ns (0.082)
TT +TC vs. CC	389 vs. 108	136 vs. 71	0.610 (0.429-0.868)	0.007	0.002	0.011
TT vs. TC+ CC	139 vs. 358	48 vs. 179	-		ns	ns
	**HW negative**	**HW positive**	**OR (IC 95%)**	**p**	**p**^a^	**p**^b^
**rs3780378**						
TT vs. TC vs. CC	145 vs. 257 vs. 118	41 vs.101 vs. 61	-	0.037	0.021	ns (0.082)
TT +TC vs. CC	402 vs. 118	142 vs. 61	0.683 (0.475-0.983)	0.044	0.016	ns (0.07)
TT vs. TC+ CC	145 vs. 375	41 vs. 172	0.655 (0.442-0.969)	0.037	0.033	ns (0.05)

^a ^Age adjusted; ^b ^Age and BMI adjusted. MS = metabolic syndrome; HTG = Hypertriglyceridemia; HW = hypertriglyceridemic waist; ns = not significant.

### Association with phenotypes and quantitative traits related to lipid metabolism

SNP rs3780378 was associated with TG values under 150 mg/dl (p = 0.013 and p = 0.006 after adjusting for age). T allele carriers showed a lower risk for HTG than the CC genotype carriers (OR = 0.61 [95% CI = 0.43-0.87]) at levels that take into account multiple testing even after correcting for covariates. Furthermore, rs3780378 was associated with HW (p = 0.037 and p = 0.021 after controlling for age). Moreover, rs3780378T carriers, assuming both a dominant and a recessive model, showed lower risk of HW with borderline nominal significance. Age-adjusted analysis confirmed a significant effect on HW risk (Table 2).

We also were able to show significant differences between rs3780378 genotypes in TG (p = 0.001), TG/HDL-C ratio (p = 0.004) and LAP (p = 0.003). However, these observations were not confirmed taking into account the effects of age (Table 3). TT genotype carriers of rs3780378 compared to allele C carriers (recessive model), showed a lower TG, LAP and TG/HDL-C ratio. Adjusted analysis confirmed the associations at levels that take into account multiple testing (Table 3). Associations under a dominant model were significant only taking into account age (data not shown).

**Table 3:** Single locus analysis of quantitative metabolic traits and surrogate measures of IR

**rs3780378**						
	**TT ( n = 186)**	**TC (n = 358)**	**CC (n = 179)**			
	Mean ± SD	Mean ± SD	Mean ± SD	p	p^a^	p^b^
**TG (mg/dL)**	120.61 ± 67.76	144.04 ± 104.745	150.17 ± 106.38	0.001 ^c^	ns	ns
**TG/HDL-C**	3.27 ± 2.60	3.99 ± 3.84	4.39 ± 4.68	0.004 ^c^	ns	ns
**LAP**	44.42 ± 37.72	54.58 ± 49.32	58.89 ± 50.77	0.003 ^c^	ns	ns
	**TT ( n = 187)**	**TC + CC (n = 537)**				
	Mean ± SD	Mean ± SD	p	p^a^	p^b^	
**TG (mg/dL)**	120.61 ± 67.76	146.09 ± 105.23	0.00017 ^c^	0.006	0.009	
**TG/HDL-C**	3.27 ± 2.60	4.13 ± 4.13	0.001 ^c^	0.008	0.011	
**LAP**	44.42 ± 37.72	56.00 ± 49.80	0.0011 ^c^	0.004	0.006	

rs3780378 recessive model. ANOVA was performed for normal-distributed data and Welch otherwise. Multiple linear regression analysis was used to adjust for possible confounding variables. Linear regression analysis was performed with log transformed values for phenotypes deviating from normality. LAP = lipid accumulation product. ^c^= Welch p value. ^a ^Age adjusted; ^b ^Age and BMI adjusted.

The association of rs3780378 genotype with TG, LAP and TG/HDL-C was not age-dependent (p = 0.059, p = 0.213 and p = 0.111 for rs3780378 genotype- by-age interaction respectively).

The SNP rs3780378 was not associated with HDL-C or LDL-C. rs7849191 was not associated with categorical or continuous variables related to lipid metabolism.

### Association with obesity and obesity-related quantitative traits

We found neither associations between both SNPs under study and obesity or abdominal obesity; nor we were able to show significant differences among genotypes in BMI, WC, fasting insulin or HOMA-IR.

### Haplotype Analyses

The inferred two-SNP-haplotype frequencies of rs7849191 and rs3780378 in that order were as follow: CC 0.18, haplotype CT 0.45, haplotype TC 0.32 and haplotype TT 0.05. In order to reduce the degree of freedom, the TT haplotype, with a frequency less than 5%, was removed from the analysis. We found 2-df association with MS (p = 0.011) when compared the overall frequency differences across three possible two-SNP- haplotypes. We could not find any other significant 2-df association. In a separate analysis, each haplotype was compared with haplotype TC. Haplotype CT (carring both protective alleles) was associated with MS even taking into account correction for multiple comparisons (CT vs. TC p = 0.00054, age adjusted p = 0.000071 and age and BMI adjusted p = 0.00010). Besides, significant difference in haplotype frequencies was found for TG values under 150 mg/dl (CT vs. TC p = 0.0069 and CC vs. TC p = 0.040) and HW (CT vs. TC; p = 0.047 and CC vs. TC p = 0.0058). Adjusted analysis confirmed these associations (TG values under 150 mg/dl: CT vs. TC age adjusted p = 0.026 and age and BMI adjusted p = 0.0055; CC vs. TC age adjusted p = 0.0023 age and BMI adjusted p = 0.068; and HW: CT vs. TC age adjusted p = 0.019 and age and BMI adjusted p = 0.047; CC vs. TC age adjusted p = 0.0029 and age and BMI adjusted p = 0.010). No association was found between the inferred two -SNP-haplotype and another obesity or lipid metabolism related phenotypes.

Two-SNP haplotype analysis of quantitative metabolic traits showed association of haplotype CT with TG (CT vs. TC p = 0.042, age adjusted p = 0.024 and age and BMI adjusted p = 0.023) and TG/HDL-C ratio (CT vs. TC p = 0.019, p = 0.044 and age and BMI adjusted p = 0.027). Association remained significant after adjustment. Association of haplotype CT with LAP became significant only after age adjustment (CT vs. TC p = 0.059, age adjusted p = 0.061 and age and BMI p = 0.017) and besides, association of haplotype CC with LAP was observed (CC vs. TC p = 0.042, age adjusted p = 0.048 and age and BMI p = 0.040). No association between another two-SNP haplotype and continuous variable related to obesity or lipid metabolism became apparent.

Although rs7849191 and rs3780378 are located in intronic regions, possible functional implications worth further investigation. The studied variants or even those that are in LD with rs3780378 may affect regulatory regions that modify the expression of genes playing roles as genetic modifiers, therefore we used the program FASTSNP [31] to investigate potential functional significance. The analysis revealed that both rs7849191 and rs3780378, and 7 SNPs in LD with rs3780378 (rs10115312, rs7034753, rs7023146, rs7043371, rs1328917, rs7857730 and rs6476939), may have a possible effect of intronic enhancer.

For identification of regulatory sequences based in evolutionarily conserved noncoding regions we performed a human/mouse whole genome comparison using the BLASTz and 'subsetAxt' programs, as described in the ensembl multicontig view help page. The results indicate that both rs7849191 and rs3780378 and at least 4 SNPs in LD (rs7037207, rs7875908, rs2230724, rs6476939) would be located in conserved regions of 1004 pb, 9977 pb, 2433 pb, 825 pb and 1411 pb respectively.

## Discussion

We examined the association between two SNPs of JAK2 and MS, phenotypes and quantitative traits related to MS in a sample of 724 unrelated men. Both rs7849191 and rs3780378 were associated with MS reduced risk at levels that take into account multiple testing. A p-value to account for multiple hypothesis testing was taken pragmatically at 0.01 as described in Statistical Analysis section. Also, we observed association of rs3780378 with both TG values under 150 mg/dl and HW; and we demonstrated differences in TG, TG/HDL-C and LAP between genotypes at levels that take into account multiple testing.

In our study we genotyped only the most significant variants of a gene wide association study conducted by Ge et al. Some results had been corroborated in our sample. The authors found that subjects homozygous for the major allele of rs3780378 had lower levels of TG in comparison to subjects homozygous or heterozygous for the minor allele; and they also failed to find association between rs3780378 and obesity related phenotypes or measurements of IR. Taking into account the results of both studies, the association between rs3780378 and TG seems to be relevant and deserves further attention. Moreover, in our sample, rs3780378 was associated with categorical and continuous variables related to TG. The authors also reported association with total cholesterol and LDL-C. Furthermore, in their cohort, rs7849191 was associated with continuous variables related to obesity. Although these associations are nominal, we should notice the poor correlation between both results. Such discrepancies are probably due to different allele frequencies or different LD structure in both populations.

We used estimated haplotype frequencies to assess the relationship between variation in a 142.8 kb region and defined phenotypes using SNP genotypes collected on cases and controls. The effect of combinations of two-SNP haplotype (rs7849191 and rs3780378 in that order) was consistent with single locus analysis. Our results show that carring both protective alleles (haplotype CT), would be associated with MS and TG values under 150 mg/dl and HW. Furthermore, haplotype analysis of quantitative metabolic traits seemed to reveal significant differences in TG, TG/HDL-C and LAP between haplotypes. Association with MS was significant at levels that take into account multiple comparisons.

The current results may be related to the role of inflammation in the development of MS and related traits such as HW, elevated TG/HDL-C and elevated LAP. Recent studies suggest that accumulation of inflammatory T-cells in adipose is an early event in obesity and MS [32,33]. We previously demonstrated in a sample selected from the same cross-sectional study, that both TG/HDL-C and LAP, were the majors predictors of MS [34] LAP and TG/HDL-C could be associated with highly lipolytic adipose tissue. Adipose tissue with high levels of lipolysis is an early and critical abnormality in the development of cardiovascular disease, DM2 and MS [35].

Pro-inflammatory cytokines negatively regulate insulin action through phosphorylation of IRS-1 or induction of SOCS expression [36]. JAK2 is the key mediator of cytokine signaling. Several studies have reported cross talk between the insulin receptor kinase and JAK signaling pathways but JAK2 involvement in insulin signaling and function remains to be investigated [37]. Besides, leptin can induce expression of SOCS3 and has been proposed as a possible mechanism for leptin resistance, since it leads to the inhibition of leptin signaling through the JAK2/STAT3 pathway.

The current study has some limitations, the sample size was relatively small and p-values were not corrected for the number of tests performed. This data should be viewed as a preliminary source for future studies including prospective studies with larger sample size and different ethnic groups.

## Conclusions

In conclusion, the present study appears to describe for the first time significant associations of common variants in JAK2 gene with MS and lipid metabolism disorders, such as HW, TG/HDL-C and LAP. These findings could be explained by the role of JAK2 in insulin and/or leptin signaling. The molecular mechanisms that explain the associations observed are still unknown but rs7849191and rs3780378 themselves, or SNPs in LD with rs3780378 may be located in regulatory regions with potential functional effects.

## Competing interests

The authors declare that they have no competing interests.

## Authors' contributions

APS and MLT carried out the molecular genetic studies, participated in the design of the study, performed the statistical analysis and drafted the manuscript. LGR and FDB carried out the biochemical analysis and contributed to the design and coordination of the study. GDF and EP designed the study, obtained samples and edited and approved the final manuscript. All authors read and approved the final manuscript.

## Pre-publication history

The pre-publication history for this paper can be accessed here:

http://www.biomedcentral.com/1471-2350/12/166/prepub
